# Therapeutic Plasma Exchange Ameliorates Incompatible Crossmatches

**DOI:** 10.4274/tjh.2016.0056

**Published:** 2016-12-01

**Authors:** Mehmet Özen, Sinan Erkul, Gülen Sezer Alptekin Erkul, Özlem Genç, Engin Akgül, Ahmet Hakan Vural

**Affiliations:** 1 Dumlupınar University Faculty of Medicine, Department of Hematology, Kütahya, Turkey; 2 Dumlupınar University Faculty of Medicine, Department of Cardiac Surgery, Kütahya, Turkey; 3 Dumlupınar University Faculty of Medicine, Blood Bank Unit, Kütahya, Turkey

**Keywords:** cardiac surgery, Apheresis, Crossmatch, Transfusion Medicine

## To the Editor,

Red blood cell (RBC) transfusion is a risk factor for mortality and morbidity in coronary artery bypass graft (CABG) surgery, and transfusion-related adverse effects may be catastrophic in these patients [1,2,3,4]. Unfortunately, there are no recommendations for these patients regarding how to proceed in the case of incompatible crossmatch tests against donors’ blood. To our knowledge, there is no report about the role of therapeutic plasma exchange (TPE) in resolving incompatible crossmatches.

**Figure 1:**
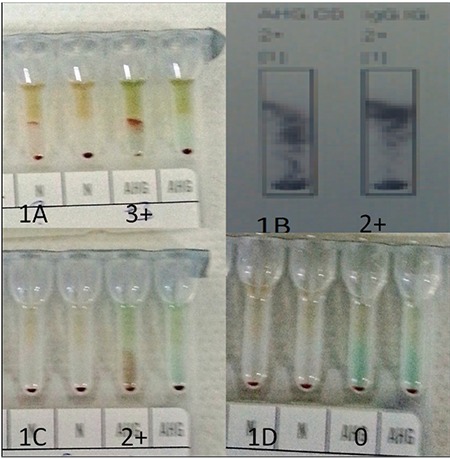
A) Crossmatch before therapeutic plasma exchange (TPE), B) direct Coombs test before TPE, C) crossmatch after one TPE, D) crossmatch after two TPEs. All tests were performed with DG gel cards (Grifols) and used the Wadiana automated blood bank (Grifols, SantCugat del Valles, Barcelona, Spain).

A 73-year-old man was admitted to our hospital because of chest pain. He had no previous medical history of coronary artery disease or any other diseases, including hemolytic disease and recent infection. In addition, he used no medication and had not received blood transfusions. After coronary angiography, a CABG was planned for the patient. Because of critical coronary artery lesions, he had to undergo the operation as soon as possible. His laboratory tests revealed mild normocytic anemia with hemoglobin of 12.8 g/dL, mean corpuscular volume of 82.2 fL, white blood cell count of 9200/µL, and platelet count of 281,000/µL. His biochemical results were normal for renal and liver function tests. The patient’s blood group was B Rh D positive based on forward and reverse grouping. Whole blood transfusion was planned for the CABG procedure by the surgeons as a part of their conventional approach. However, cross match results revealed 3+ reactions against B Rh D positive donors’ whole blood and other B Rh D positive RBCs in the blood bank (Figure 1A). Direct Coombs test results were 2+ AHG and IgG (Figure 1B). Due to the urgency of the planned CABG, we did not wait for detailed antibody screening test results, and TPE (Infomed, Geneva, Switzerland) was performed. Total body plasma was exchanged with fresh frozen plasma within 2 h. After one TPE procedure, the cross-reaction to donors’ whole blood was 2+. TPE was performed again 1 day later, and after the second TPE, the crossmatches were compatible (Figures 1C and 1D). There was no adverse effect due to TPE. We operated after the second TPE, used a regular erythrocyte suspension and whole blood, administered 40 mg/day intravenous methylprednisolone for 4 days, and discharged the patient 1 week after the operation. Two weeks after the operation, he had no hematological or antibody-related disease and he had a normal complete blood count with compatible crossmatches. He also had no antibodies related to incompatible crossmatches.

In a patient undergoing CABG, an incompatible blood transfusion can lead to perioperative hemolysis and increased mortality [5,6]. Defining the antibodies and finding compatible blood for a patient with incompatible crossmatches can be a challenging and time-consuming problem [5,7].

TPE is an important treatment modality for many autoimmune conditions and helps by removing autoantibodies [8]. Our patient did not have time to wait and needed CABG urgently. Therefore, we assumed that the patient had antibody-related autoimmune hemolytic anemia and treated him with TPE. We report that this approach may be efficient for patients with incompatible crossmatch results even if they do not have autoimmune hemolytic anemia. Therefore, TPE might be reserved for urgent conditions or when identification of antibodies is inconclusive.
